# 

**Published:** 1917-02-10

**Authors:** 


### AN APPROPRIATE GIFT.

SOMETHING typically appropriate in the way of a gift is recorded by the Medical Superintendent of the Leeds Corporation. He reports the gift of three stones of sugar to the hospitals by Mr. J. Fowler, of Wellington Bridge, as a thankoffering for the treatment of his son in one of the hospitals.

### Matrons' and Sisters' Appointments.

Tuberculosis Sanatorium, Birtley, co. Durham.—Miss Hannah Edmonds has been appointed matron. She was trained at the Infirmary and Broomlands Children's Hospital, Middlesbrough, and has since held the post of ward sister and holiday matron at the Cameron Hospital, West Hartlepool, and deputy matron at the County Borough Sanatorium, Sunderland.

Wirral Fever Hospital, Clatterbridge.—Miss Jessie Baker has been appointed matron. She was trained at Liverpool Stanley Hospital and has since been matron of the Isolation Hospitals at Sittingbourne and Devonport.

Royal Infirmary, Sheffield.—Miss M. C. Moss has been appointed home sister and assistant matron. She was trained at St. Mary's Hospital, Paddington, where she has since held the post of ward sister and night superintendent.

Borough Hospital, Birkenhead.—Miss Katherine Rowland has been appointed night sister. She was trained at the Royal Infirmary, Liverpool, and has since held the post of sister at the Children's Hospital, Birkenhead; the Royal Southern Hospital, Liverpool; and the Children's Hospital, Leasowe.

Children's Hospital, Lower Sydenham.—Miss Olive Smith has been appointed theatre sister. She was trained at the City of Westminster Infirmary, Fulham Road, and later held the post of ward sister at the Children's Infirmary, Cleveland Street, W.

General Infirmary, Peterborough.—Miss Lucy Pridham has been appointed sister. She was trained at the General Infirmary, Leeds, where she was later staff nurse.

Liverpool Maternity Hospital.—Miss A. M. Sanders has been appointed sister. She was trained at the Bethnal Green Infirmary, and has been surgical and theatre sister at the Sanatorium, Watford, and at Dover Military Hospital. She is a member of Q.A.I.M.N.S.R.

National Hospital for Diseases of the Heart.—Miss Grace Dews has been appointed night sister. She was trained at St. George's-in-the-East Infirmary, and later became ward sister and sister of the midwifery department. Miss Dews has also been sister-in-charge of Harbury Isolation Hospital, and sister at Broadstairs Red Cross Military Hospital.

Putney Hospital.—Miss Jessie Alison McRitchie has been appointed sister. She was trained at Arbroath Infirmary, where she has since been temporary sister. She has also been staff nurse at Bolingbroke Hospital.

Red Cross Auxiliary Hospital, Chaseside, St. Anne's-on-Sea.—Miss G. Mary Thwaites has been appointed sister. She was trained at Blackburn and East Lancashire Royal Infirmary, and has since held appointments at the Victoria Hospital, Keighley; the Victoria Hospital, Romford; and at Gravesend Hospital.

Red Cross Hospital, Llandaff.—Mrs. McKenzie has been appointed sister. She was previously sister at the Hamadryad Seamen's Hospital, Cardiff.

Reigate and Redhill General Hospital, Redhill.—Miss M. F. Lund has been appointed night sister. She was trained at Great Yarmouth General Hospital, where she was afterwards night sister, and she also had two years' training at Ipswich Borough Isolation Hospital.

Willesden Institution, Acton Lane, N.W.—Miss Annie E. Durrant has been appointed superintendent night charge nurse. She was trained at Greenwich Union Infirmary, where she was later ward sister, a position which she also held at the North-Eastern Hospital, Tottenham More lately Miss Durrant has been assistant superintendent nurse at the Derby Union Infirmary.

**NOTE.—Particulars of Appointments for insertion in this column will be welcomed.**

### PASSING EVENTS AND TOPICS.

### Pound Day at Guildford.

"Pound Day" at the Royal Surrey County Hospital resulted in 5,402 lb. of commodities being sent in.

### A Doll Auction.

The annual doll auction at Brighton in aid of the Royal Alexandra Hospital for Sick Children and the Sussex County Hospital realised £170.

### A Newcastle Corporation Scheme.

In co-operation with other authorities in Northumberland and Durham, the Newcastle Corporation has adopted a scheme for dealing with venereal diseases at a cost of over £10,000 a year.

### Calcutta's Gift to Scottish Women's Hospital.

A cablegram received at the headquarters in Edinburgh of the Scottish Women's Hospital states that Calcutta has presented the hospitals with a donation of £13,000.

### Guardians Appeal for Economy.

The Fulham Board of Guardians have made an appeal for more economy on the part of the eighteen visiting doctors at the Fulham Military Hospital. It was pointed out that quite one quarter of the medical men's monthly cheques represented travelling expenses, they being allowed 6d. per mile for the use of a four-seater motor and 4d. per mile for a three-seater. The Chairman urged the doctors to reduce their travelling expenses by using the trams and 'buses, which, he said, would bring them to the hospital as quickly as their own cars.

### Stoke Newington's Cot at Alton.

For a Stoke Newington cot at Lord Mayor Treloar's Hospital £270 has been raised.

### A Medicine Fund.

Mrs. Jacks, widow of Mr. William Jacks, LL.D., of Kelvinside, Glasgow, has left £5,000 to be held in trust for the purpose of maintaining a nurse for the sick and poor of the Callander district and for providing medicines and comforts for the sick poor.

### Royal College of Surgeons of England.

The Hunterian Lecture is to be delivered in the Theatre of the College on Monday, February 12, 1917, at 5 p.m., by Professor J. Hutchinson, F.R.C.S. The subject is "Dupuytren," and the lecture will deal with "Dupuytren's Contraction," the history and development of the operative treatment since Dupuytren's time, the drawbacks of the methods now usually employed; the reasons for their not infrequent failure, and with Dupuytren's life and surgical works.

### Name of a State Hospital Changed.

The name of the Long Island State Hospital for the Insane has been changed to the Brooklyn State Hospital. The change is of very great advantage for the avoidance of confusion with the Long Island College Hospital, which is situated in the heart of Brooklyn, near the east shore of New York Harbour. The two hospitals are situated in widely different parts of the city, and there should no longer be trouble over the misdirection of mails, packages, or patients.

Mr. John Milne Smith, of Cheltenham, who died on November 20, left the residue of his estate for division into twenty parts (each part not to exceed £500), and directed that four shares should go to the Royal Alexandra Infirmary, Paisley.

RATES OF SUBSCRIPTION: Twelve months, 6/6; Six months, 4/-; Three months, 2/-; post free to any address in the United Kingdom. Abroad, Twelve months, 8/8; Six months, 5/-; Three months, 2/6, post free. THE HOSPITAL "Index" to Volume LX., April to September 1916, is now ready, price 2½d. per copy, post free. Address The Manager, "THE HOSPITAL," The Hospital Building, 28/29 Southampton Street, Strand, London, W.C.

